# Cystathionine β‐synthase is required for oocyte quality by ensuring proper meiotic spindle assembly

**DOI:** 10.1111/cpr.13322

**Published:** 2022-08-19

**Authors:** Yan Cao, Xinyu Zhu, Panpan Zhen, Ying Tian, Dengyu Ji, Ke Xue, Wenjing Yan, Jiayin Chai, Huirong Liu, Wen Wang

**Affiliations:** ^1^ Department of Physiology and Pathophysiology School of Basic Medical Sciences, Capital Medical University Beijing China; ^2^ Department of Pathology Beijing Luhe Hospital, Capital Medical University Beijing China; ^3^ Department of Histology and Embryology School of Basic Medical Sciences, Capital Medical University Beijing China; ^4^ Beijing Key Laboratory of Metabolic Disorders Related Cardiovascular Diseases Capital Medical University Beijing China

## Abstract

**Objectives:**

Poor oocyte quality is detrimental to fertilization and embryo development, which causes infertility. Cystathionine β‐synthase (CBS) is one of the key enzymes modulating the metabolism of homocysteine (Hcy). Studies have shown that CBS plays an important role in female reproduction. However, the role of CBS in regulating oocyte quality during meiotic maturation still needs further investigation.

**Materials and Methods:**

Immunohistochemistry, immunofluorescence, drug treatment, western blot, cRNA construct and in vitro transcription, microinjection of morpholino oligo and cRNA were performed for this study.

**Results:**

We found that CBS was expressed both in human and mouse oocytes of follicles. In mouse oocytes, CBS was distributed in the nucleus at germinal vesicle (GV) stage and localized to spindle from germinal vesicle breakdown (GVBD) to metaphase II (MII). The expression of CBS was reduced in ovaries and oocytes of aged mice. CBS depletion resulted in meiotic arrest, spindle abnormality and chromosome misalignment, disrupted kinetochore‐microtubule attachments and provoked spindle assembly checkpoint (SAC). CBS was disassembled when microtubules were disrupted with nocodazole, and co‐localized with the stabilized microtubules after taxol treatment. Furthermore, CBS depletion decreased the acetylation of α‐tubulin.

**Conclusions:**

These results reveal that CBS is required for the acetylation of α‐tubulin to ensure proper spindle assembly in regulating oocyte quality during meiotic maturation.

## Graphical abstract

CBS is required for oocyte quality by ensuring proper spindle assembly. During oocyte meiotic maturation, CBS ensures spindle assembly by participating in the acetylation of α‐tubulin to stabilize microtubules. Kinetochores remain attached by spindle microtubules. SAC is removed from the kinetochores and then the chromosomes can be separated correctly.

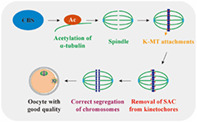

## 1 INTRODUCTION

Poor oocyte quality means the incapability of oocyte to go through successful maturation, fertilization and embryo development, causing infertility.^1^ During meiotic maturation, proper spindle assembly is essential to the correct segregation of chromosomes.^2^ Poor oocyte quality with increasing age remains an important issue for female fertility.^3^

The level of serum homocysteine (Hcy) is increased in some infertile patients with poor quality oocytes.^4^ It is well known that cystathionine β‐synthase (CBS) is the key enzyme modulating the metabolism of Hcy, which catalyzes Hcy with serine or cysteine into cystathionine and water or hydrogen sulfide (H_2_S).^5^, ^6^, ^7^, ^8^ It has been also reported that the expression of CBS in the liver of infertile rats is reduced, which partly explains the increased serum Hcy level.^4^ Until now it is not clear whether the expression of CBS is reduced in oocytes with poor quality. In recent years, increasing evidence has indicated that CBS is important for female reproduction.^9^, ^10^ It has been shown that CBS is expressed in mouse follicular cells.^11^ However, whether it is expressed in human or mouse oocytes has not been reported yet. The inhibition of CBS in follicular cells hinders oocyte meiotic maturation, as shown by the increased rate of GV oocytes.^12^ The role of CBS in regulating oocyte quality during meiotic maturation still needs further exploration.

In this study, we aim to explore the role of CBS in regulating oocyte quality during meiotic maturation and focus on the underlying mechanism in meiotic spindle assembly by participating in acetylation of α‐tubulin.

## 2 MATERIALS AND METHODS

### 2.1 Oocyte collection and culture

All the procedures of animal care and use were in accordance with Animal Care Commission policies of Capital Medical University. Female CB6F1 mice (21–23 days) were injected with 10 IU pregnant mare serum gonadotropin (PMSG) to stimulate pre‐ovulatory follicle development.^13^, ^14^ BALB/C female mice were injected with 10 IU PMSG.^15^, ^16^ The mice were euthanatized with CO_2_ and ovaries were transferred to MEM (1×) + GlutaMAX‐I (Gibco) medium. Follicles in ovaries were punctured with a sterile injection needle. Cumulus‐oocyte complexes (COCs) were released and collected into new MEM (1×) + GlutaMAX‐I. The COCs were incubated in MEM Alpha (1×) (Gibco) containing 3 mg/ml bovine serum albumin (BSA, MRC) and 10% fetal bovine serum (FBS, Corning) for 0, 2, 4, 8, 12, and 17 h at 37°C with 5% CO_2_. The oocytes developed to GV, GVBD, pre‐metaphase I (Pre‐MI), metaphase I (MI), anaphase/telephase I (AI/TI) and MII. Then cumulus cells in COCs were removed, while oocytes with full size, normal perivitelline space, modest zona pellucida^17^, ^18^ were collected for drug treatment, microinjection, immunofluorescence, western blot and so on.

### 2.2 HL‐7702 culture

HL‐7702 cell line was cultured in DMEM (BI) including 10% FBS at 37°C with 5% CO_2_.

### 2.3 Antibodies

Rabbit polyclonal anti‐CBS antibody was purchased from Proteintech (Cat#: 14787‐1‐AP); mouse monoclonal anti‐CBS antibody was purchased from Santa Cruz Biotechnology (Cat#: sc‐133154); mouse monoclonal anti‐acetylated α‐tubulin (lysine‐40) antibody was purchased from Sigma (Cat#: T7451); human polyclonal anti‐CREST antibody was purchased from Antibodies Incorporated (Cat#:15‐234‐0001); mouse monoclonal anti‐MAD1 antibody was purchased from Santa Cruz Biotechnology (Cat#: sc‐137025); mouse monoclonal anti‐Lamin A antibody was purchased from Abcam (Cat#: ab8980); mouse monoclonal anti‐Alpha tubulin antibody was purchased from Proteintech (Cat#: 66031‐1‐Ig); rabbit polyclonal anti‐GAPDH antibody was purchased from Sigma (Cat#: SAB4300645); Alexa Fluor 488‐conjugated goat anti‐mouse IgG (H + L) (Cat#: ZF‐0512), Alexa Fluor 594‐conjugated goat anti‐rabbit IgG (H + L) (Cat#: ZF‐0516), FITC‐conjugated goat anti‐human IgG (H + L) (Cat#: ZF‐0308), HRP‐conjugated goat anti‐rabbit IgG (H+L) (Cat#: ZB‐2301), HRP‐conjugated goat anti‐mouse IgG (H+L) (Cat#: ZB‐2305) were purchased from Zhongshan Golden Bridge Biotechnology.

### 2.4 Immunohistochemistry

Human ovarian sections containing normal follicles were collected from patients with ovarian mature teratoma, ovarian seromucinous cystadenoma, luteal cyst, yolk sac tumor and immature teratoma in the Department of Pathology, Luhe Hospital, Capital Medical University. We obtained informed consent for experiments with human subjects and complied with the privacy rights of human subjects. Mouse ovary sections were available from 7‐week‐old female BALB/C mice. Sections were dewaxed in xylene and hydrated in graded alcohol (100%–70%). Endogenous peroxidase was blocked in 3% H_2_O_2_ for 30 min. Antigen retrieval was performed in citrate buffer (pH 6) at 100°C for 2 min. The sections were washed in phosphate buffer saline (PBS, Sigma) and non‐specific binding was reduced in 5% BSA for 1 h. The sections were incubated with primary antibody (anti‐CBS antibody 1: 500) at 4°C overnight. Ovary sections were washed and incubated with secondary antibody for 1 h. Diaminobenzidine (DAB) and haematoxylin staining were performed. The sections were dehydrated and mounted in neutral resin.

### 2.5 Immunofluorescence

Oocytes were fixed in PEM‐buffer (100 mM Pipes, pH 6.9, 1 mM MgCl_2_, 1 mM EGTA) with 1% paraformaldehyde (PFA, Sigma) and 0.5% TritonX‐100 (Sigma) for 1 h at room temperature and then rinsed in PBS with 0.2% TritonX‐100. Non‐specific binding sites were blocked in PBS supplemented with 1% BSA, 2.28% glycine, 0.2% TritonX‐100, and 10% normal goat serum (NGS, Zhongshan Golden Bridge Biotechnology) for 1 h at room temperature. Then oocytes were incubated with primary antibodies at 4°C overnight. Oocytes were washed in PBS with 0.2% TritonX‐100 and then incubated with secondary antibodies for 1 h at room temperature. After being washed in PBS with 0.2% TritonX‐100, the oocytes were mounted on glass slides in VECTASHIELD mounting medium with 4′,6‐diamidino‐2‐phenylindole (DAPI, Vector Laboratories) and imaged by an upright fluorescent microscope (Olympus Microsystems). The fluorescence intensity of the samples was analyzed by Image J software (National Institutes of Health).

HL‐7702 was fixed in 4% PFA for 20 min and permeabilized with 0.1% Triton X‐100 for 30 min. After being blocked in 1% BSA for 1 h, cells were incubated in primary antibody (anti‐CBS antibody 1: 500) at 4°C overnight and incubated in secondary antibody for 1 h. HL‐7702 was mounted on glass slides in mounting medium with DAPI and examined using a confocal microscopy (Leica).

Human ovarian sections were dewaxed with xylene and hydrated in graded alcohol for immunofluorescence. Fluorescence was imaged using an upright fluorescent microscope (Olympus Microsystems).

### 2.6 Drug treatment

MI oocytes were treated with 20 mg/ml nocodazole (Sigma‐Aldrich) for 15 min or 10 μM taxol (Selleck) for 40 min in MEM (1×) + GlutaMAX‐I at 37°C. Oocytes in control group were treated with the same concentration of dimethyl sulfoxide (DMSO, AppliChem). The oocytes were fixed for immunofluorescence.

### 2.7 Western blot

Oocytes were lysed in Laemmli sample buffer (BIO‐RAD) with β‐mercaptoethanol (Sigma) and protease inhibitor cocktail (Sigma) and boiled for 5 min. Then they were separated on 10% SDS‐PAGE gel at 120 V for 1.5 h, transferred to the polyvinylidene fluoride (PVDF, Millipore) membrane at 400 mA for 1 h, and washed in Tris‐buffered saline with 0.1% Tween 20 (TBST). Non‐specific blots were blocked in TBST containing 5% skimmed milk for 1 h at room temperature and washed in TBST. The membrane was incubated in primary antibody at 4°C overnight and then washed in TBST. PVDF membrane was transferred to secondary antibody for 1 h at room temperature and then washed in TBST. The blots were covered with appropriate amount of ECL Plus Substrate (Thermo Scientific). The images were captured by the gel documentation system and relative signal intensities of immunoreactive bands were analyzed using gel software Image Lab 3.0.

### 2.8 Depletion of CBS by microinjection of *Cbs* specific morpholino oligo

The COCs were cultured at 37°C with 5% CO_2_ for 1 h in the MEM alpha (1×) medium containing 3.33 μM milrinone (Sigma) and 10% FBS to be arrested at GV stage. The GV‐intact oocytes were microinjected with 10 pl of 1 mM *Cbs* morpholino oligo (5′‐ATTTTCAGAGGGAGCGAAGACCT‐3′, Gene Tools) to knockdown CBS expression or 10 pl of 1 mM standard control oligo (5′‐CCTCTTACCTCAGTTACAATTTATA‐3′, Gene Tools) as a Control group. GV oocytes without microinjection were designated as the Uninjected group. Oocytes were cultured for 24 h in M16 medium (Sigma) containing 3.33 μM milrinone and washed in milrinone‐free MEM (1×) + GlutaMAX‐I. Then they were transferred to M16 medium without milrinone and incubated for 0, 8, and 17 h for immunofluorescence and western blot.

### 2.9 Cold treatment

Oocytes were cultured for 8 h in M16 medium and then treated in MEM (1×) + GlutaMAX‐I on ice for 5 min for immunofluorescence.

### 2.10 cRNA construct and in vitro transcription

*Cbs* cDNA was sub‐cloned into pUC57/Myc vector. *TubK40Q* or *TubK40R* cDNA was sub‐cloned into pcDNA3.1/Myc vector. Plasmids were extracted using TIANprep Mini Plasmid Kit (TIANGEN) and linearized by HindIII (NEB). cRNA was synthesized using HiScribe T7 ARCA mRNA kit (with tailing) (NEB) and purified with Monarch RNA Cleanup Kit (NEB). GV‐intact oocytes were injected with 1.0 μg/μl cRNA for further experiments.

### 2.11 Statistical analysis

The data repeated at least three independent experiments were presented as the mean ± standard error of mean (SEM). Statistical differences were evaluated by *t* test or one‐way analysis of variance (ANOVA) using GraphPad Prism 5 software (GraphPad Software) and *p* < 0.05 was considered significant.

## 3 RESULTS

### 3.1 CBS was expressed both in human and mouse oocytes

**FIGURE 1:**
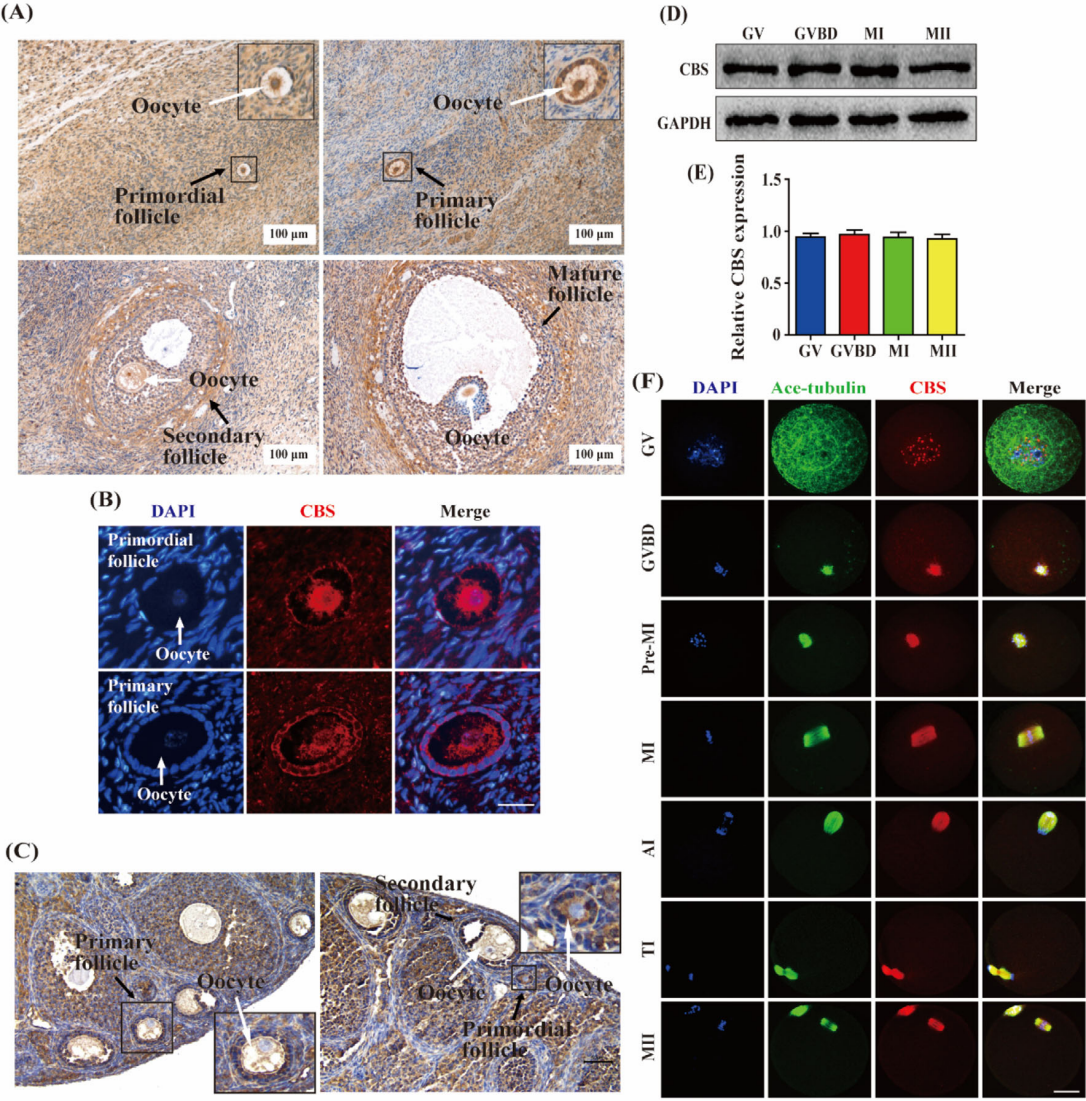
Cystathionine β‐synthase (CBS) was expressed both in human and mouse oocytes. (A) Human ovarian sections were collected for immunohistochemistry with CBS antibody. Scale bar, 100 μm. The black arrow referred to follicles and the white arrow referred to oocytes. (B) Human ovarian sections were collected for immunofluorescence with CBS antibody (red). The white arrow referred to oocytes. Scale bar, 20 μm. (C) Immunohistochemistry for CBS in sections of mouse ovary. Scale bar, 50 μm. The black arrow referred to follicles and the white arrow referred to oocytes. (D) Mouse oocytes were cultured for 0, 2, 8, 17 h and developed to germinal vesicle (GV), germinal vesicle breakdown (GVBD), metaphase I (MI), metaphase II (MII) stages for western blot. Protein samples were probed with CBS and glyceraldehyde‐3‐phosphate dehydrogenase (GAPDH) antibody, respectively. (E) Data were expressed as mean ± SEM of at least three independent experiments. GV: *n* = 108, GVBD: *n* = 108, MI: *n* = 108, MII: *n* = 108. *p* > 0.05. (F) Mouse oocytes were cultured for 0, 2, 4, 8, 12, 17 h and developed to GV, GVBD, pre‐metaphase I (Pre‐MI), MI, anaphase/telephase I (AI/TI), MII stage for immunofluorescence with CBS antibody (red) and anti‐acetylated α‐tubulin (Ace‐tubulin) antibody (green). Scale bar, 20 μm

In order to determine whether CBS was expressed in human oocytes, immunohistochemistry and immunofluorescence were performed with human ovarian sections. Our results showed that CBS was expressed in oocytes of primordial follicles, primary follicles, secondary follicles, and mature follicles (Figure 1A,B). In addition, we also found that CBS was expressed in mouse oocytes of primordial follicles, primary follicles and secondary follicles by immunohistochemistry (Figure 1C), which was consistent with the distribution pattern of CBS in human oocytes of follicles. Then, we used mice as a model for further study. Western blot showed a high level of CBS protein from GV to MII stage (Figure 1D,E). Immunofluorescence revealed that CBS was punctately localized in the nucleus at GV stage. Strikingly, at MI stage CBS was distributed in the cytoplasm, similar to the spindle shape (Figure S1). GV oocytes developed to GVBD, Pre‐MI, MI, AI/TI, MII stage, and CBS was colocalized with the spindle microtubules (Figure 1F), suggesting that CBS may be involved in meiotic spindle formation.

HL‐7702 was selected for immunofluorescence to explore the distribution of CBS during mitosis. In HL‐7702, CBS was uniformly distributed in the cytoplasm during interphase, prophase, metaphase, anaphase and telophase (Figure S2), which was different from the distribution in oocytes and suggested CBS may play a different role in meiosis and mitosis.

### 3.2 Expression of CBS was reduced in ovaries and oocytes of aged mice

**FIGURE 2:**
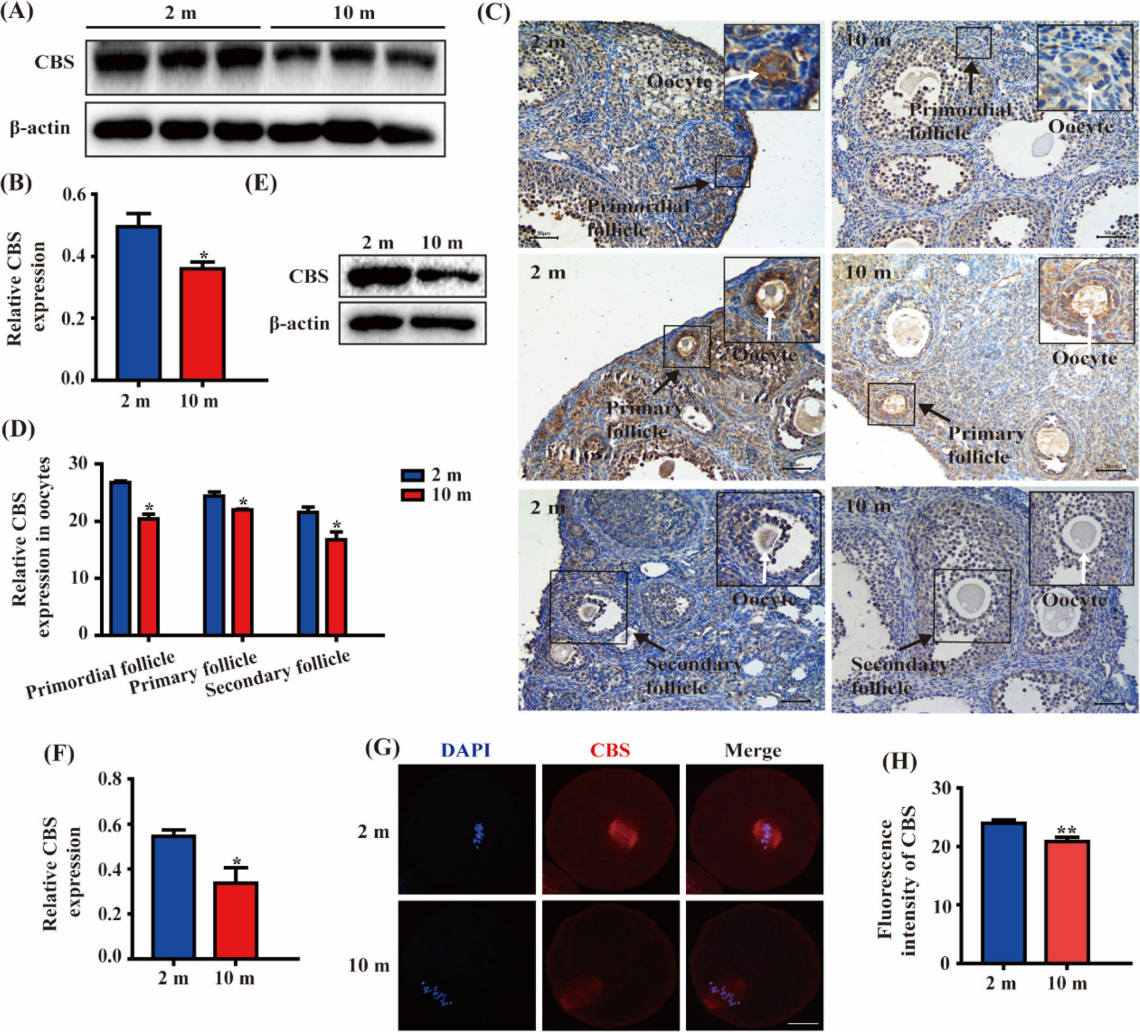
The expression of CBS was reduced in ovaries and oocytes of 10‐month‐old mice. (A) Western blot showed the level of CBS protein in ovaries of 10‐month‐old mice was reduced. (B) Data were expressed as mean ± SEM, 2 m: *n* = 9, 10 m: *n* = 9. *p < 0.05. (C) The expression of CBS in 10‐month‐old mouse oocytes of follicles was decreased, presented by immunohistochemistry. Scale bar, 50 μm. The black arrow referred to follicles, the white arrow referred to oocytes. (D) Data were expressed as mean ± SEM of at least three independent experiments. 2 m: *n* = 3, 10 m: *n* = 3. *p < 0.05. (E) Western blot showed the expression of CBS in MI oocytes of 10‐month‐old mice was decreased. (F) Data were expressed as mean ± SEM of at least three independent experiments. 2 m: *n* = 99, 10 m: *n* = 99. *p < 0.05. (G) MI oocytes were removed from 2‐ and 10‐month‐old mice for immunofluorescence with CBS antibody (red). Scale bar, 20 μm. (H) Data were expressed as mean ± SEM of at least three independent experiments. 2 m: *n* = 25, 10 m: *n* = 22. **p < 0.01

To investigate the relationship between the CBS and oocytes with poor quality, the ovaries of 2‐ and 10‐month‐old female mice were selected for western blot and immunohistochemistry. We observed that the expression of CBS was reduced in ovaries (Figure 2A,B) and oocytes (Figure 2C,D) of 10‐month‐old mice. Next, oocytes were removed from ovaries to develop to MI stage for further detection. Both western blot (Figure 2E,F) and immunofluorescence (Figure 2G,H) displayed the reduced expression of CBS in oocytes of 10‐month‐old mice, indicating that CBS may be related with oocyte quality regulation.

### 3.3 Depletion of CBS impaired meiotic progression

**FIGURE 3:**
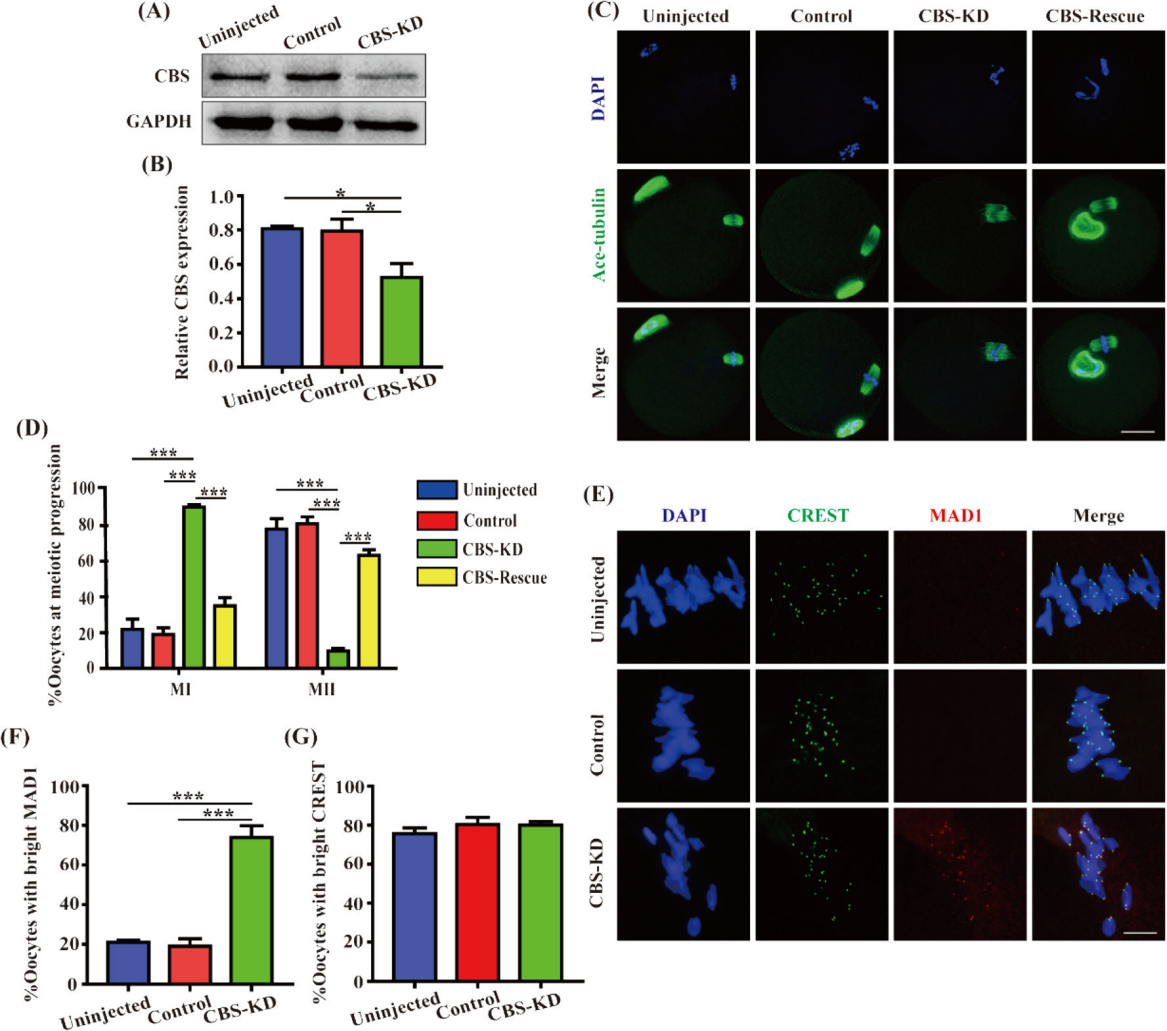
Depletion of CBS led to the poor quality of oocytes. (A) GV oocytes uninjected or injected with 10 pl of 1 mM control or *Cbs* morpholino were incubated in M16 medium with 3.3 μM milrinone for 24 h and then GV oocytes were collected for western blot. Protein samples were probed with CBS antibody and GAPDH antibody, respectively. (B) Data were expressed as mean ± SEM of at least three independent experiments. Uninjected: *n* = 114, Control: *n* = 114, CBS‐KD: *n* = 114. *p < 0.05. (C) Uninjected, Control, CBS‐depleted GV oocytes or CBS‐depleted GV oocytes injected with *Cbs‐Myc* cRNA were washed in MEM (1×) + GlutaMAX‐I for 3 times instantly and cultured in M16 medium for 17 h to MII for immunofluorescence with acetylated α‐tubulin antibody (green). Scale bar, 20 μm. (D) Data were expressed as mean ± SEM of at least three independent experiments. Uninjected: *n* = 58, Control: *n* = 49, CBS‐KD: *n* = 54, CBS‐Rescue: *n* = 56. ***p < 0.001. (E) Uninjected, Control, CBS‐KD oocytes were cultured in M16 medium for 8 h to MI for immunofluorescence with Mitotic arrest deficiency 1 (MAD1) (red) and human anti‐centromere antibody (CREST) (green). Scale bar, 20 μm. (F) Data were expressed as mean ± SEM of at least three independent experiments. Uninjected: *n* = 38, Control: *n* = 30, CBS‐KD: *n* = 34. ***p < 0.001. (G) Data were expressed as mean ± SEM of at least three independent experiments. Uninjected: *n* = 38, Control: *n* = 30, CBS‐KD: *n* = 34. *p* > 0.05

In order to further evaluate the role of CBS in oocyte quality regulation during meiotic maturation, specific *Cbs* morpholino was utilized to knock down the expression of CBS protein. Western blot revealed CBS was significantly reduced in CBS‐depleted oocytes (Figure 3A,B). Meanwhile, the fluorescence intensity of CBS was decreased (Figure S3A,B), indicating that CBS was successfully depleted by *Cbs* morpholino. GV oocytes were injected with *Cbs‐Myc* cRNA. CBS‐Myc and overexpression of CBS were detected in CBS‐OE oocytes (Figure S4), demonstrating exogenous CBS protein was successfully expressed in mouse oocytes. The oocytes were cultured for 17 h to observe meiotic progression. As shown in Figure 3C,D, the first polar body extrusion (PBE) rate was markedly decreased in CBS‐depleted oocytes and most of them were arrested at MI stage. Furthermore, exogenous administration of CBS in CBS‐depleted oocytes can reverse this phenomenon, suggesting that CBS was associated with oocyte quality regulation during meiotic maturation. Jia et al. have reported that exogenous Hcy leads to the decrease of the PBE rate in porcine oocytes and impairs oocyte quality.^19^ In order to verify whether the poor quality caused by CBS depletion in mouse oocytes was due to the elevated Hcy, the culture medium and lysed oocytes were collected to detect the level of Hcy by enzyme‐linked immunosorbent assay (ELISA). The results showed that there was no significant difference in Hcy level either in culture medium or lysed oocytes (Figure S5A,B), implying the role of CBS in mouse oocyte quality regulation does not depend on the elevation of Hcy. Meiotic arrest in CBS‐depleted oocytes implied that the spindle assembly checkpoint (SAC) was activated. Oocytes were immunostained with the antibody of MAD1, a component of SAC and oocytes in CBS‐KD group had brighter MAD1 (Figure 3E,F), indicating CBS depletion provoked the SAC. Furthermore, there was no significant difference in CREST signal between Uninjected, Control, and CBS‐depleted oocytes (Figure 3G).

### 3.4 Depletion of CBS impaired spindle assembly and disrupted kinetochore‐microtubule attachments

**FIGURE 4:**
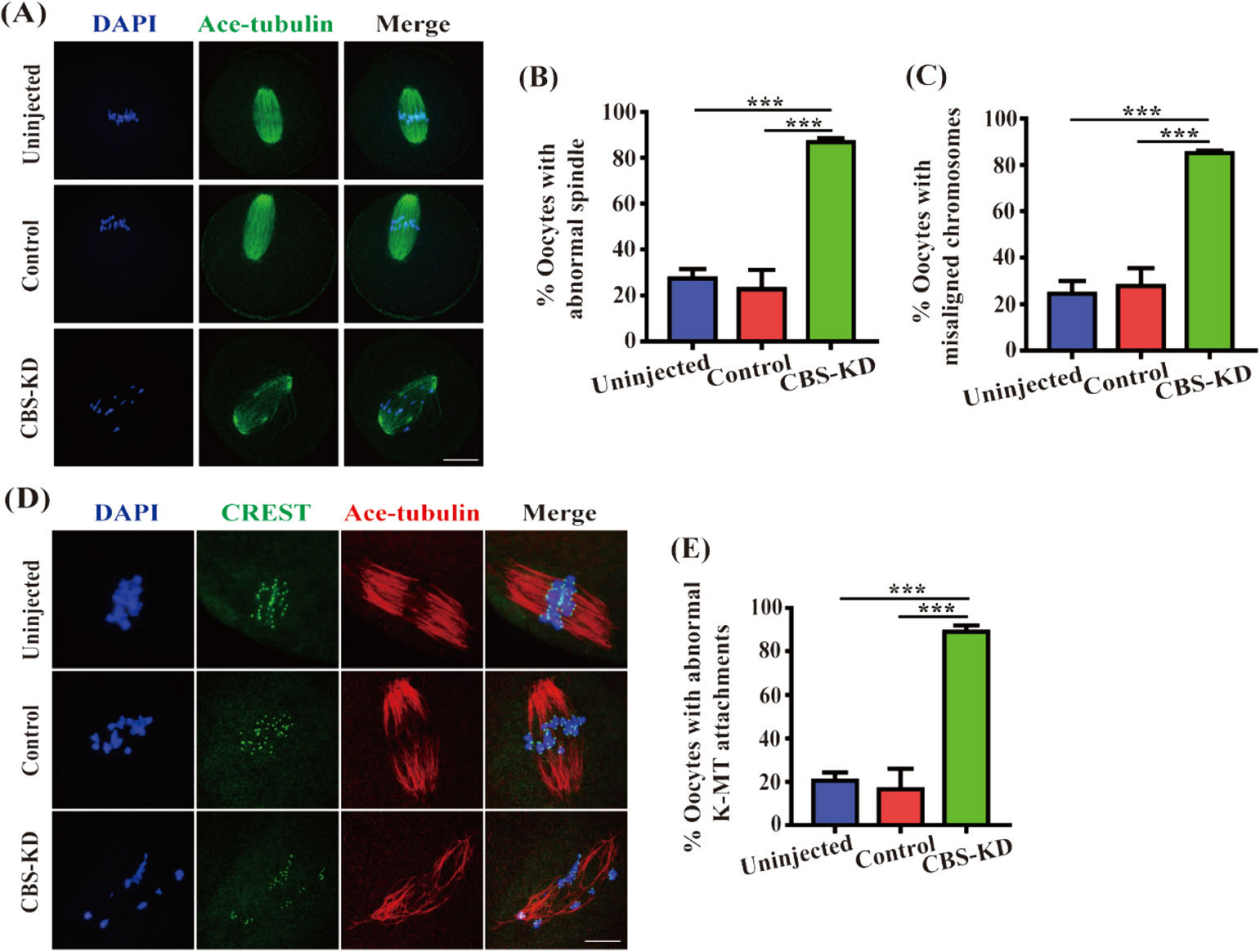
Abnormal spindle and compromised kinetochore‐microtubule (K‐MT) attachments in CBS‐depleted oocytes at MI. (A) Oocytes in Uninjected, Control, CBS‐KD groups were cultured in M16 medium for 8 h to MI for immunofluorescence with acetylated α‐tubulin antibody (green). Scale bar, 20 μm. (B, C) Data were expressed as mean ± SEM of at least three independent experiments. Uninjected: *n* = 64, Control: *n* = 62, CBS‐KD: *n* = 63. ***p < 0.001. (D) Oocytes in Uninjected, Control, CBS‐KD groups were cultured in M16 medium for 8 h to MI and then incubated on ice for 5 min and processed for immunofluorescence with acetylated α‐tubulin (red) and CREST antibody (green). (E) Data were expressed as mean ± SEM of at least three independent experiments. Uninjected: *n* = 53, Control: *n* = 50, CBS‐KD: *n* = 58. ***p < 0.001

We next assessed the spindle morphologies and chromosome alignment after injection. Characteristic barrel‐shape MI spindles with the well‐aligned chromosomes were observed in Uninjected and Control oocytes. However, CBS‐KD oocytes showed a number of disorganized spindles and misaligned chromosomes (Figure 4A–C), demonstrating CBS was essential for meiotic spindle assembly. Spindle disorganization and chromosome misalignment suggested defective kinetochore‐microtubule (K‐MT) attachments in CBS‐depleted oocytes. In Uninjected and Control oocytes, kinetochores remained attached by spindle microtubules after cold treatment for 5 min. However, the depletion of CBS destroyed the attachments between microtubule and kinetochore (Figure 4D,E).

### 3.5 CBS was required for acetylation of α‐tubulin in oocytes

**FIGURE 5:**
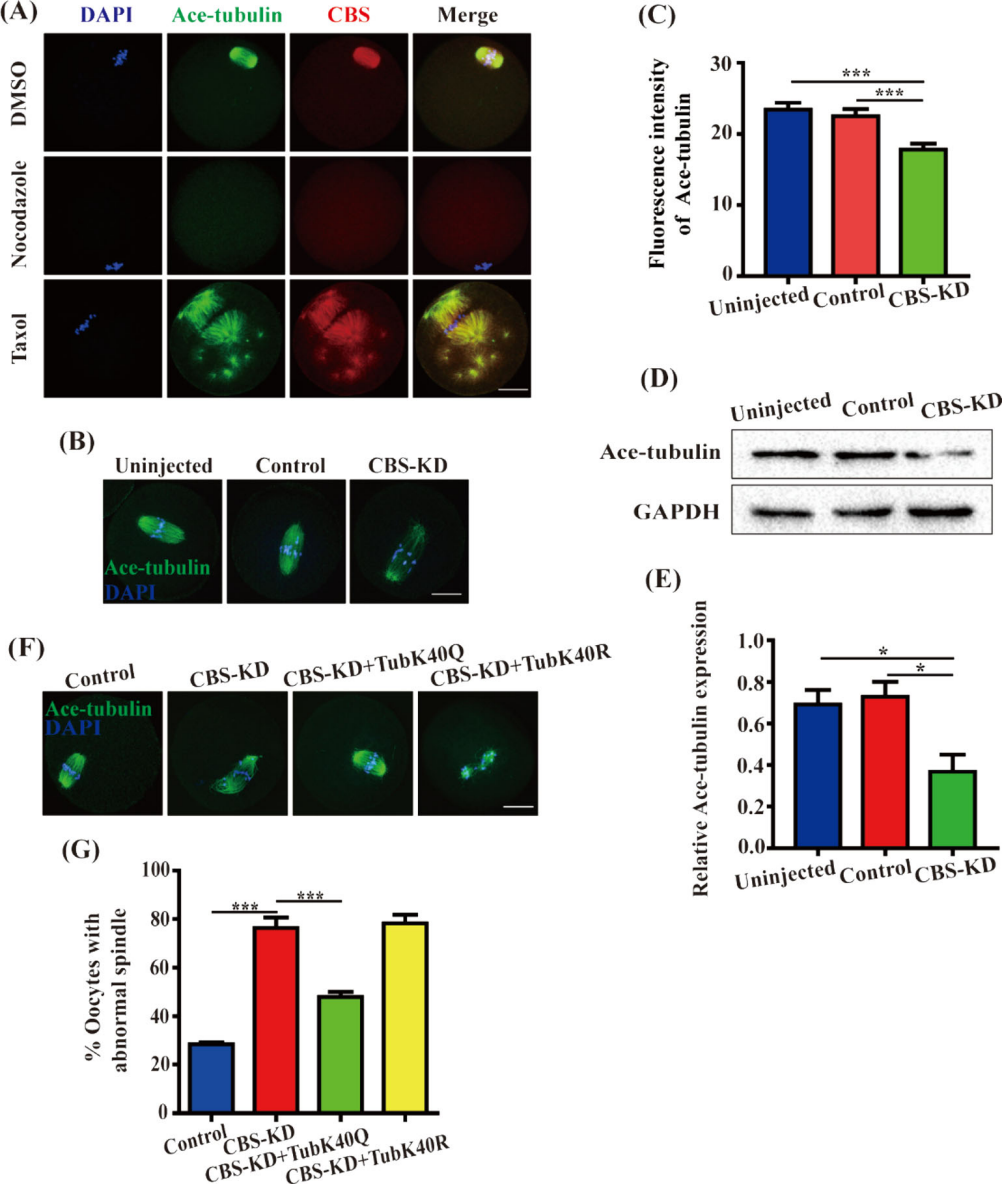
CBS was required for acetylation of α‐tubulin. (A) Oocytes were cultured for 8 h to MI and then treated with nocodazole for 15 min, or taxol for 40 min in MEM (1×) + GlutaMAX‐I for immunofluorescence with acetylated α‐tubulin (green) and CBS antibody (red). Scale bar, 20 μm. (B) Fluorescence intensity of acetylated α‐tubulin (green) in MI oocytes of Uninjected, Control, CBS‐KD groups. Scale bar, 20 μm. (C) Data were expressed as mean ± SEM of at least three independent experiments. Uninjected: *n* = 44, Control: *n* = 41, CBS‐KD: *n* = 43. ***p < 0.001. (D) MI oocytes in Uninjected, Control and CBS‐KD groups were collected for western blot. Protein samples were probed with acetylated α‐tubulin and GAPDH antibody, respectively. (E) Data were expressed as mean ± SEM of at least three independent experiments. Uninjected: *n* = 117, Control: *n* = 117, CBS‐KD: *n* = 117. *p < 0.05. (F) Control, CBS‐depleted GV oocytes or CBS‐depleted GV oocytes injected with *TubK40Q‐Myc* or *TubK40R‐Myc* cRNA were washed in MEM (1×) + GlutaMAX‐I for 3 times instantly and cultured in M16 medium for 8 h to MI for immunofluorescence with acetylated α‐tubulin antibody (green). Scale bar, 20 μm. (G) Data were expressed as mean ± SEM of at least three independent experiments. Control: *n* = 57, CBS‐KD: *n* = 56, CBS‐KD + TubK40Q: *n* = 48, CBS‐KD + TubK40R: *n* = 52. ***p < 0.001

Abnormal spindle assembly urged us to assess the role of CBS in the microtubule stability. MI oocytes were treated with a microtubule‐depolymerizing drug nocodazole and we found as microtubules were depolymerized, the CBS signal localized to the spindle disappeared completely. After treatment with a microtubule‐stabilizing reagent taxol, broad spindles were observed in MI oocytes, CBS was co‐localized with spindle microtubules (Figure 5A), which suggested that the subcellular localization of CBS was related with microtubule dynamics. The acetylation level of a‐tubulin on lysine 40 (K40) has been reported to be a marker of stabilized microtubules.^20^ CBS‐depleted oocytes showed a decreased fluorescence intensity of acetylated α‐tubulin (Figure 5B,C), implying CBS may ensure spindle assembly by participating in the acetylation of α‐tubulin to stabilize microtubules. In order to further verify this hypothesis, oocytes were lysed for western blot with acetylated α‐tubulin, the results revealed that the expression of acetylated α‐tubulin was remarkably decreased in CBS‐KD oocytes (Figure 5D,E). In addition, the fluorescence intensity of α‐tubulin was not significantly different in CBS‐depleted oocytes (Figure S6). Next, to confirm whether the abnormal spindle was due to the defective acetylation of α‐tubulin, *TubK40Q‐Myc* or *TubK40R‐Myc* cRNA was injected into CBS‐depleted oocytes. As displayed in Figure 5F,G, it was TubK40Q acetylmimic mutant not TubK40R nonacetylatable mutant that rescued the abnormal spindle, indicating that CBS was required for spindle assembly by participating in the acetylation of α‐tubulin to stabilize microtubules.

## 4 DISCUSSION

In this study, we demonstrated CBS was expressed both in human and mouse oocytes. In mouse oocytes, CBS was co‐localized with spindle microtubules after meiosis resumption. The expression of CBS was decreased in ovaries and oocytes of aged mice. Depletion of CBS led to meiotic arrest, disrupted the spindle assembly by the defective acetylation of α‐tubulin, destroyed the kinetochore‐microtubule attachments and provoked SAC.

Infertility has become a public health problem worldwide.^21^ With the change of people's lifestyle, the incidence of infertility is gradually increasing.^22^ It has been reported that the rate of fertility all over the world will decline year by year before 2050.^23^ Oocyte quality is a key factor to pregnancy success.^24^ However, the molecular mechanism involved in regulating oocyte quality during meiotic maturation still needs further studies. CBS is a key enzyme in metabolizing Hcy, which catalyzes the formation of cystathionine and water or H_2_S from Hcy with serine or cysteine.^8^ In recent years, it has been found that CBS has an important role in female reproduction.^9^, ^10^, ^11^, ^12^ The role of CBS in oocyte quality regulation during meiotic maturation still needs to be further explored.

We found that CBS was expressed in human oocytes of primordial follicles, primary follicles, secondary follicles, and mature follicles. As 99% of human genes have mice homologues, mice were generally used in reproductive research.^25^, ^26^ Immunohistochemistry showed CBS was also expressed in mouse oocytes of primordial follicles, primary follicles and secondary follicles, which is similar to the distribution of CBS in human oocytes of follicles. Therefore, we used mice as a research model for the next discussion. We found stable expression of CBS during meiotic maturation. The localization of CBS to spindle from GVBD to MII stage implied that CBS may be involved in meiotic spindle formation. Meanwhile in HL‐7702, different from that in oocytes, CBS was uniformly distributed in the cytoplasm, which implied that CBS may play a different role in meiosis and mitosis. For women, after 30 years old, oocyte quality is gradually decreased along with age.^27^, ^28^ Compared with 2‐month‐old female mice, the oocyte quality of 10‐month‐old female mice is significantly decreased.^29^ Our data showed the CBS expression in ovaries and oocytes was decreased in aged mice. Specificity protein 1 (Sp1) is a transcription factor binding to *CBS* promoter.^30^ Zimon et al. have reported that the expression of Sp1 is decreased in the ovaries of aged mice,^31^ which partly explains the decreased expression of CBS in our data. Reactive oxygen species (ROS) is increased during ovarian aging.^32^ Treatment with H_2_O_2_ increases phosphorylation level of Sp1 in a human alveolar epithelial cell line (HAE).^33^ Wu et al. have reported in rat kidney, increased phosphorylation of Sp1 results in the decrease of transcriptional activity during ischemia/reperfusion, which leads to a reduction of CBS protein.^30^ In aged ovaries, the increase of ROS may increase phosphorylation of Sp1 leading to decreased transcriptional activity, which partly explains the decreased expression of CBS in our study. Therefore, CBS was reduced in ovaries and oocytes of aged mice, which may be due to the decreased expression and increased phosphorylation of Sp1. The decreased expression of CBS in aged oocytes indicated CBS may be related with oocyte quality regulation. The perivitelline space is higher in oocytes of *Cbs* knockout mice,^34^ suggesting that the quality of these oocytes is low^35^ and CBS may be involved in oocyte quality regulation. To further confirm the role of CBS in oocyte quality regulation during meiotic maturation, specific *Cbs* morpholino was used to knock down CBS protein. The depletion of CBS impaired meiotic progression. Moreover, most oocytes were arrested at MI stage and exogenous CBS can reverse this phenomenon, indicating depletion of CBS led to the decline of oocyte quality during meiotic maturation. Interestingly, in our data, there was no significant difference in the Hcy level in either the culture medium or the total oocytes, implying that the poor quality of mouse oocytes caused by CBS depletion may be not related with the increase of Hcy.

MI oocytes developing to AI is inseparable from the correct segregation of chromosomes. When correct attachments are established between the spindle microtubule and the kinetochore, mitotic arrest deficient protein 2 (Mad2) is removed from the kinetochore and cell division cycle protein 20 (Cdc20) is released from mitotic checkpoint complex (MCC). Anaphase promoting complex/cyclosome (APC/C) is activated and then securin and Cyclin B are degraded. After that, separase cleaves the cohesion between chromosome arms. Then homologous chromosomes are separated.^36^, ^37^, ^38^ Therefore, any factor that impacts spindle formation may hinder the correct segregation of chromosomes, leading to meiotic arrest. A great quantity of disorganized spindles and misaligned chromosomes in CBS‐depleted oocytes demonstrated that CBS was involved in meiotic spindle assembly. Moreover, depletion of CBS destroyed the K‐MT attachments and provoked SAC. Oocytes treated with nocodazole and taxol showed CBS was related with microtubule dynamics. In our study, IF and western blot showed the fluorescence intensity and expression of acetylated α‐tubulin were decreased in CBS‐depleted oocytes. TubK40Q is a mutant of α‐tubulin, in which lysine 40 is substituted with glutamine to mimic acetylated tubulin. TubK40R is also a mutant of α‐tubulin, in which lysine 40 is substituted with arginine to mimic nonacetylated tubulin.^39^, ^40^ In our study, it was TubK40Q acetylmimic mutant not TubK40R non‐acetylatable mutant that rescued the abnormal spindle, demonstrating that CBS was essential for spindle assembly by participating in the acetylation of α‐tubulin. It has been reported that histone deacetylase 6 (HDAC6) and sirtuin 2 (SIRT2) are the deacetylases of α‐tubulin K40. α‐tubulin acetyltransferase 1 (α‐TAT1) is the acetylase of α‐tubulin K40.^41^ Li et al. have reported histone deacetylase 3 (HDAC3) can modulate the acetylation of ɑ‐tubulin.^42^ Maybe the depletion of CBS can impair the acetylation modification of α‐tubulin by affecting some of the above‐mentioned deacetylases and acetylase, which needs further effort to be examined in the future. In summary, CBS is required for the acetylation of α‐tubulin to ensure spindle assembly in oocyte quality regulation during meiotic maturation. In aged oocytes, the acetylation levels of tubulin are elevated.^43^ It has been reported the expression of both HDAC3 and SIRT2 is reduced in aged oocytes, which results in hyperacetylation of α‐tubulin in these aged oocytes.^43^, ^44^ The elevated acetylation levels of tubulin in aged oocytes may be due to hyperacetylation of α‐tubulin by the reduced expression of HDAC3 and SIRT2 being stronger than hypoacetylation by the reduced CBS expression.

Altogether, CBS is involved in oocyte quality regulation during meiotic maturation. CBS can participate in the acetylation of α‐tubulin to ensure proper spindle assembly, kinetochore‐microtubule attachments and the removal of SAC from the kinetochores. Eventually, chromosomes are correctly segregated. Consequently, our findings will provide a theoretical reference for improving oocyte quality.

## AUTHOR CONTRIBUTIONS

Yan Cao, Huirong Liu, and Wen Wang designed the project. Yan Cao, Xinyu Zhu, Ying Tian, Dengyu Ji, Ke Xue, Wenjing Yan, and Jiayin Chai performed the experiments. Yan Cao and Panpan Zhen collected human ovarian sections. Yan Cao and Wen Wang analyzed the data. Yan Cao and Wen Wang wrote the manuscript.

## CONFLICT OF INTEREST

The authors declare no conflict of interest.

## Supporting information


**SUPPLEMENTARY FIGURE 1**. The distribution of CBS in mouse oocytes. The mouse oocytes were obtained and cultured for 0 h, 8 h and developed to GV, MI stage for immunofluorescence. CBS was represented in red and DAPI was represented in blue. Scale bar, 20 μm.
**SUPPLEMENTARY FIGURE 2**. The distribution of CBS in HL‐7702. HL‐7702 was cultured for immunofluorescence with acetylated α‐tubulin (green) and CBS antibody (red). Scale bar, 20 μm.
**SUPPLEMENTARY FIGURE 3**. Fluorescence intensity of CBS was significantly decreased in CBS‐depleted oocytes at MI. (A) Oocytes in Uninjected, Control, CBS‐KD groups were cultured in M16 medium for 8 h to MI for immunofluorescence with CBS antibody (red). Scale bar, 20 μm. (B) Data were expressed as mean ± SEM of at least three independent experiments. Uninjected: *n* = 50, Control: *n* = 46, CBS‐KD: *n* = 52. ***p < 0.001.
**SUPPLEMENTARY FIGURE 4**. The overexpression of CBS in GV oocytes. (A) Protein samples were probed with Myc, CBS and GAPDH antibody, respectively. (B) Data were expressed as mean ± SEM of at least three independent experiments. Control: *n* = 105, CBS‐OE: *n* = 105. ***p < 0.001. (C) Data were expressed as mean ± SEM of at least three independent experiments. Control: *n* = 105, CBS‐OE: *n* = 105. **p < 0.01.
**SUPPLEMENTARY FIGURE 5**. There was no significant difference in homocysteine (Hcy) level either in culture medium or in total oocytes at MI stage. (A) Culture medium of Uninjected, Control, and CBS‐depleted oocytes at MI stage were collected to detect Hcy by enzyme‐linked immunosorbent assay (ELISA). Data were expressed as mean ± SEM of at least three independent experiments. Uninjected: *n* = 66, Control: *n* = 66, CBS‐KD: *n* = 66. *p* > 0.05. (B) Uninjected, Control, CBS‐depleted oocytes at MI stage were lysed by repeated freezing and thawing at −80°C to detect Hcy by ELISA. Data were expressed as mean ± SEM of at least three independent experiments. Uninjected: *n* = 45, Control: *n* = 45, CBS‐KD: *n* = 45. *p* > 0.05.
**SUPPLEMENTARY FIGURE 6**. The fluorescence intensity of α‐tubulin was not significantly different between Uninjected, Control and CBS‐KD oocytes. (A) Oocytes in Uninjected, Control, CBS‐KD groups were cultured in M16 medium for 8 h to MI for immunofluorescence with α‐tubulin antibody (green). Scale bar, 20 μm. (B) Data were expressed as mean ± SEM of at least three independent experiments. Uninjected: *n* = 16, Control: *n* = 20, CBS‐KD: *n* = 21. *p* > 0.05.

## Data Availability

The data needed to support the results of this study are available from the corresponding author upon reasonable request.
