# Supplementary material for: Cystathionine β‐synthase is required for oocyte quality by ensuring proper meiotic spindle assembly
Source: Cell Prolif. 2022 Aug 19;55(12):e13322. doi: 10.1111/cpr.13322 (PMC9715357; doi:10.1111/cpr.13322)
Supplement: Supplementary file 1 — SUPPLEMENTARY FIGURE 1. The distribution of CBS in mouse oocytes. The mouse oocytes were obtained and cultured for 0 h, 8 h and developed to GV, MI stage for immunofluorescence. CBS was represented in red and DAPI was represented in blue. Scale bar, 20 μm. SUPPLEMENTARY FIGURE 2. The distribution of CBS in HL‐7702. HL‐7702 was cultured for immunofluorescence with acetylated α‐tubulin (green) and CBS antibody (red). Scale bar, 20 μm. SUPPLEMENTARY FIGURE 3. Fluorescence intensity of CBS was significantly decreased in CBS‐depleted oocytes at MI. (A) Oocytes in Uninjected, Control, CBS‐KD groups were cultured in M16 medium for 8 h to MI for immunofluorescence with CBS antibody (red). Scale bar, 20 μm. (B) Data were expressed as mean ± SEM of at least three independent experiments. Uninjected: n = 50, Control: n = 46, CBS‐KD: n = 52. ***p < 0.001. SUPPLEMENTARY FIGURE 4. The overexpression of CBS in GV oocytes. (A) Protein samples were probed with Myc, CBS and GAPDH antibody, respectively. (B) Data were expressed as mean ± SEM of at least three independent experiments. Control: n = 105, CBS‐OE: n = 105. ***p < 0.001. (C) Data were expressed as mean ± SEM of at least three independent experiments. Control: n = 105, CBS‐OE: n = 105. **p < 0.01. SUPPLEMENTARY FIGURE 5. There was no significant difference in homocysteine (Hcy) level either in culture medium or in total oocytes at MI stage. (A) Culture medium of Uninjected, Control, and CBS‐depleted oocytes at MI stage were collected to detect Hcy by enzyme‐linked immunosorbent assay (ELISA). Data were expressed as mean ± SEM of at least three independent experiments. Uninjected: n = 66, Control: n = 66, CBS‐KD: n = 66. p > 0.05. (B) Uninjected, Control, CBS‐depleted oocytes at MI stage were lysed by repeated freezing and thawing at −80°C to detect Hcy by ELISA. Data were expressed as mean ± SEM of at least three independent experiments. Uninjected: n = 45, Control: n = 45, CBS‐KD: n = 45. p > 0.05. SUPPLEMENTARY FIGURE 6. T [file CPR-55-e13322-s001.docx]

Supplementary material

**Supplementary Figure 1.**


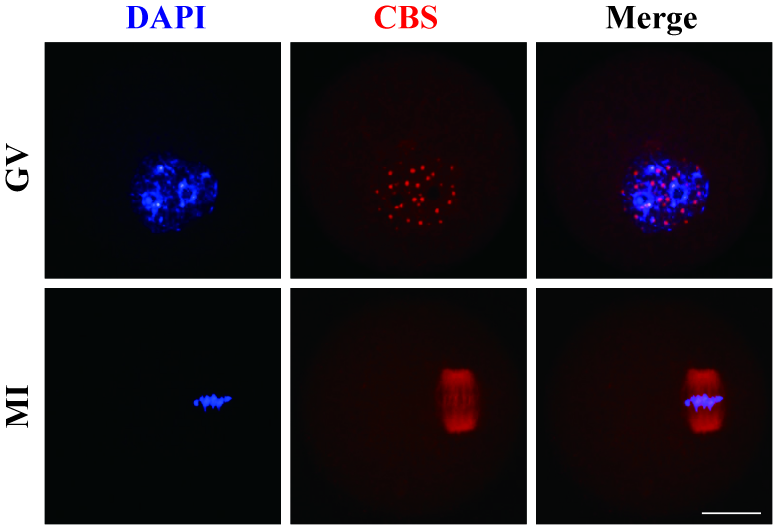


**Supplementary Figure 1** The distribution of CBS in mouse oocytes. The mouse oocytes were obtained and cultured for 0 h, 8 h and developed to GV, MI stage for immunofluorescence. CBS was represented in red and DAPI was represented in blue. Scale bar, 20 μm.

**Supplementary Figure 2.**


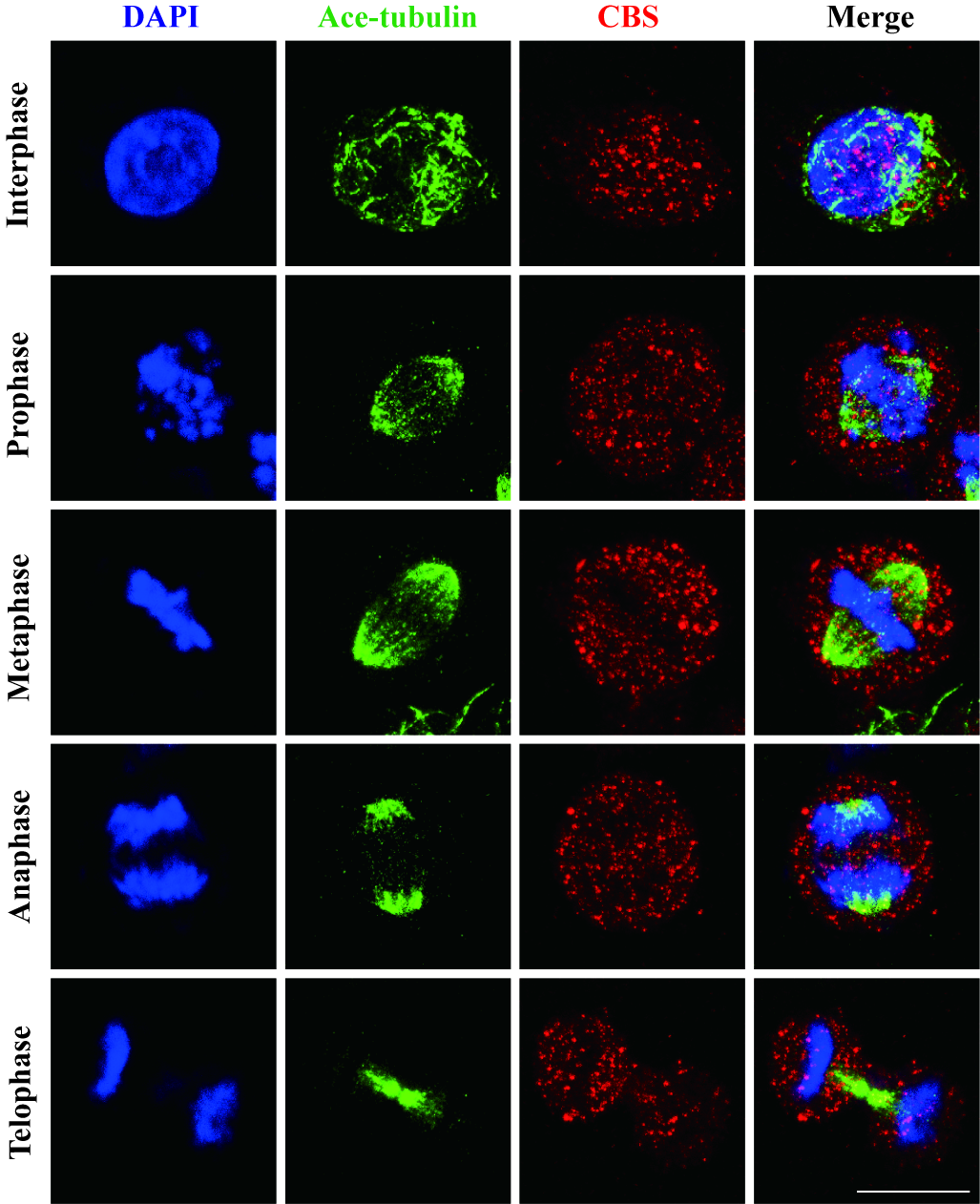


**Supplementary Figure 2** The distribution of CBS in HL-7702. HL-7702 was cultured for immunofluorescence with acetylated α-tubulin (green) and CBS antibody (red). Scale bar, 20 μm.

**Supplementary Figure 3.**


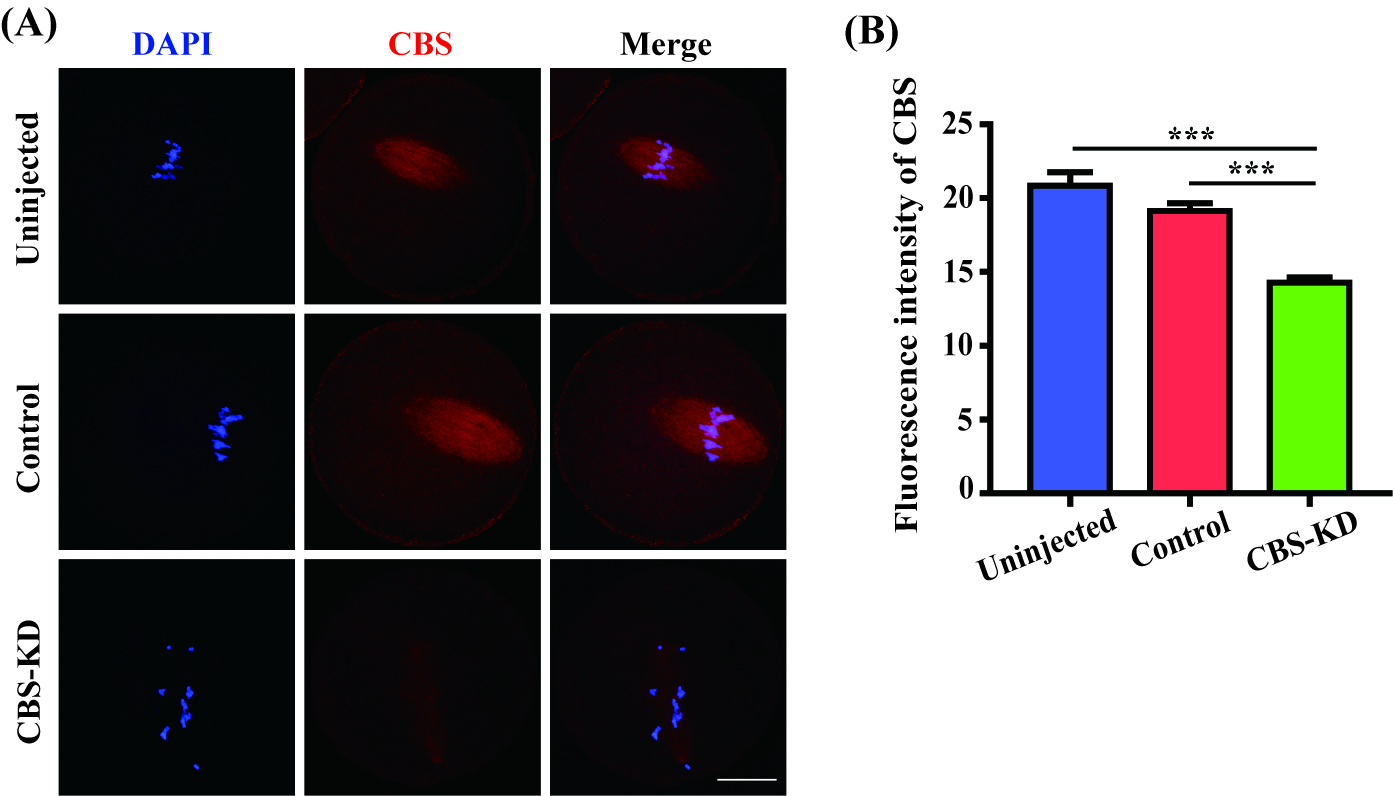


**Supplementary Figure 3** Fluorescence intensity of CBS was significantly decreased in CBS-depleted oocytes at MI. (A) Oocytes in Uninjected, Control, CBS-KD groups were cultured in M16 medium for 8 h to MI for immunofluorescence with CBS antibody (red). Scale bar, 20 μm. (B) Data were expressed as mean ± SEM of at least three independent experiments. Uninjected: n=50, Control: n=46, CBS-KD: n=52. ****P*<0.001.

**Supplementary Figure 4.**


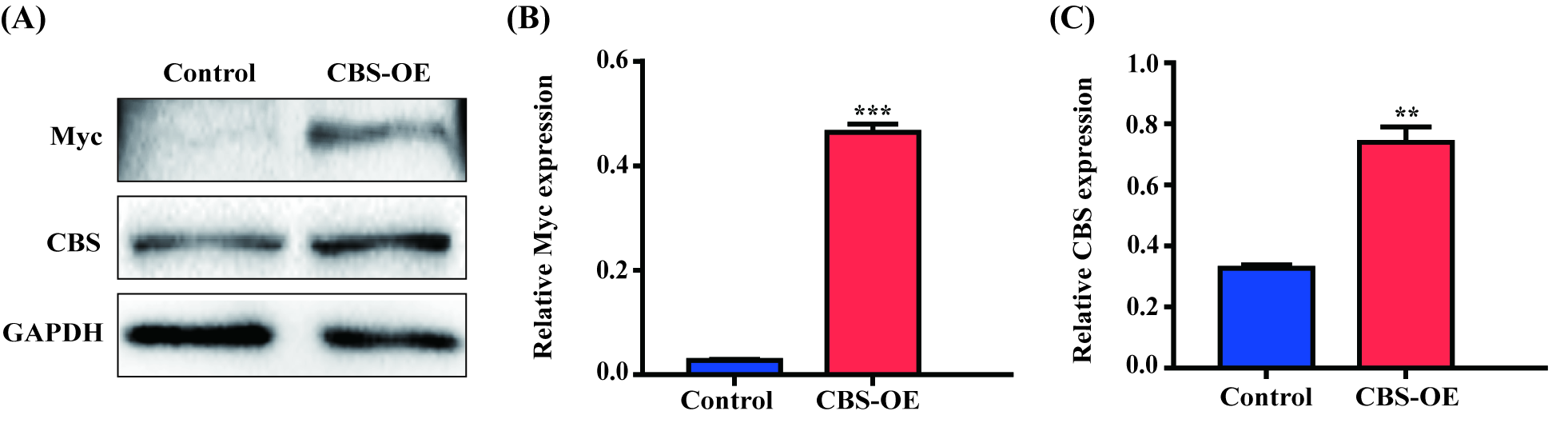


**Supplementary Figure 4** The overexpression of CBS in GV oocytes. (A) Protein samples were probed with Myc, CBS and GAPDH antibody, respectively. (B) Data were expressed as mean ± SEM of at least three independent experiments. Control: n=105, CBS-OE: n=105. ****P*<0.001. (C) Data were expressed as mean ± SEM of at least three independent experiments. Control: n=105, CBS-OE: n=105. ***P*<0.01.

**Supplementary Figure 5.**


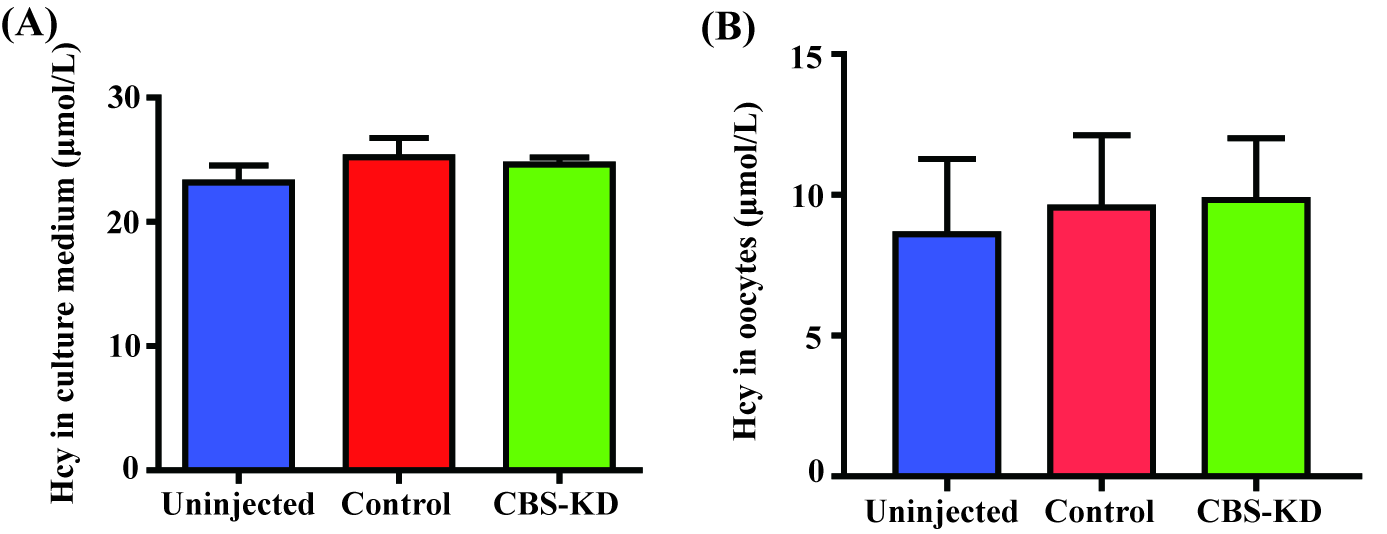


**Supplementary Figure 5** There was no significant difference in homocysteine (Hcy) level either in culture medium or in total oocytes at MI stage. (A) Culture medium of Uninjected, Control, and CBS-depleted oocytes at MI stage were collected to detect Hcy by enzyme-linked immunosorbent assay (ELISA). Data were expressed as mean ± SEM of at least three independent experiments. Uninjected: n=66, Control: n=66, CBS-KD: n=66. *P* > 0.05. (B) Uninjected, Control, CBS-depleted oocytes at MI stage were lysed by repeated freezing and thawing at -80℃ to detect Hcy by ELISA. Data were expressed as mean ± SEM of at least three independent experiments. Uninjected: n=45, Control: n=45, CBS-KD: n=45. *P* > 0.05.

**Supplementary Figure 6.**


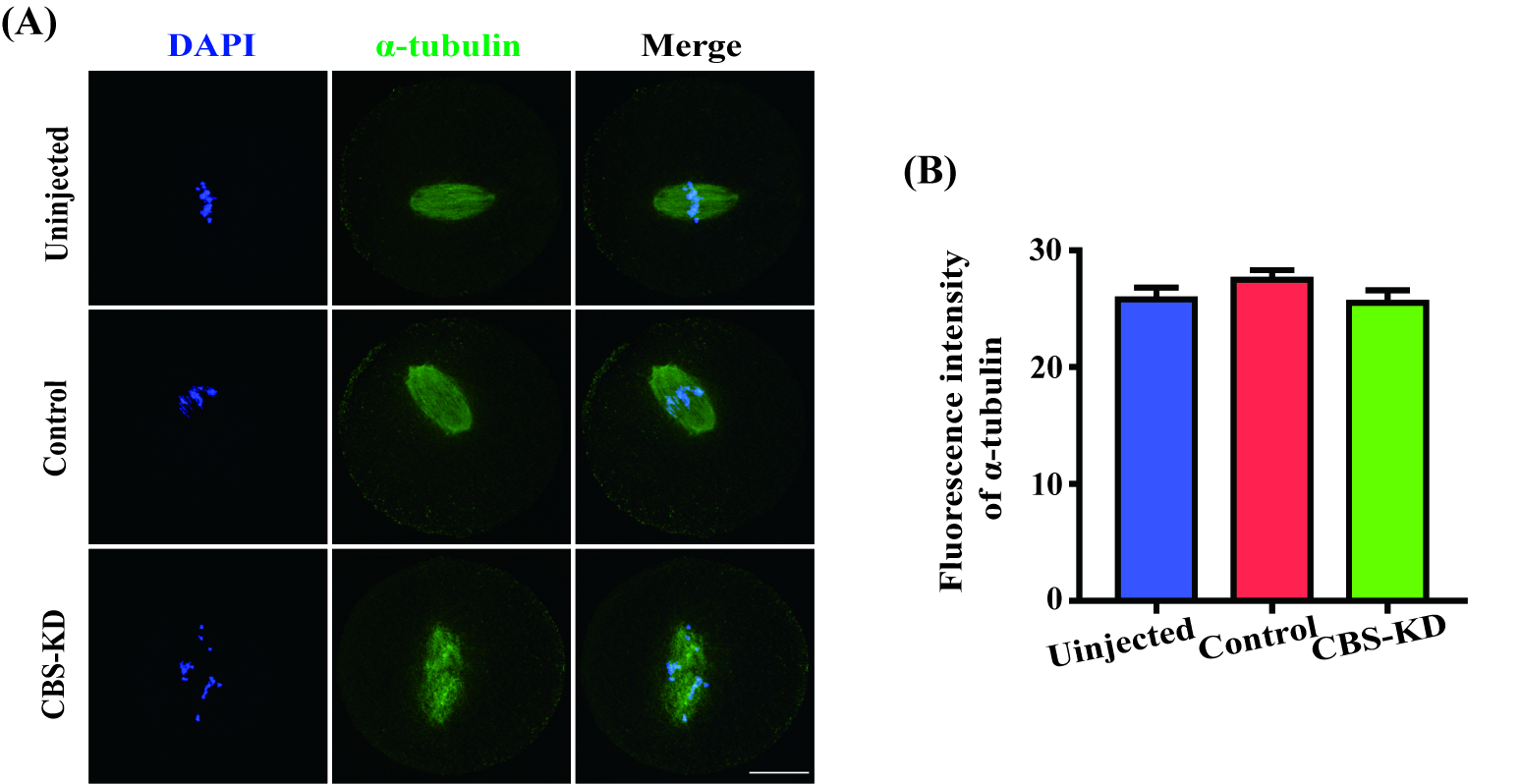


**Supplementary Figure 6** The fluorescence intensity of α-tubulin was not significantly different between Uninjected, Control and CBS-KD oocytes. (A) Oocytes in Uninjected, Control, CBS-KD groups were cultured in M16 medium for 8 h to MI for immunofluorescence with α-tubulin antibody (green). Scale bar, 20 μm. (B) Data were expressed as mean ± SEM of at least three independent experiments. Uninjected: n=16, Control: n=20, CBS-KD: n=21. *P* > 0.05.
